# Changes of serum CA125 and PGE2 before and after high-intensity focused ultrasound combined with GnRH-a in treatment of patients with adenomyosis

**DOI:** 10.1515/med-2023-0794

**Published:** 2024-03-05

**Authors:** Yan Huang, Yuzhen Zhou, Huixian Chen, Yanyi Xu

**Affiliations:** Department of Obstetrics and Gynecology, Suzhou Hospital of Integrated Traditional Chinese and Western Medicine, Suzhou, Jiangsu, 215101, China

**Keywords:** CA125, PGE2, HIFU, GnRH-a

## Abstract

This study aimed to investigate the changes of serum carbohydrate antigen 125 (CA125) and prostaglandin E2 (PGE2) in patients with adenomyosis before and after treatment with high-intensity focused ultrasound (HIFU) combined with gonadotropin-releasing hormone agonist (GnRH-a). One hundred and sixty-five patients with adenomyosis who received HIFU combined with GnRH-a were selected as case group. Sixty-five healthy women who underwent physical examination at the same time were taken as normal control group. At the end of follow-up 6 months after treatment, the case group were divided into effective subgroup and ineffective subgroup according to clinical efficacy. Changes of serum CA125 and PGE2 were analyzed. Serum CA125 and PGE2 levels in the case group were higher than those in the normal control group before treatment (both *P* < 0.001). Serum CA125 and PGE2 levels in the case group 6 months after treatment were lower than those before treatment (both *P* < 0.001). There was no difference in serum CA125 and PGE2 levels between effective subgroup and ineffective subgroup before treatment (*P* = 0.351, 0.284, respectively). Serum CA125 and PGE2 levels in the effective subgroup were lower than those in the ineffective subgroup 6 months after treatment (both *P* < 0.001). Serum CA125 and PGE2 may be involved in the development of adenomyosis, and their expression levels may be related to the prognosis of patients. Levels of serum CA125 and PGE2 in patients with adenomyosis decrease after treatment with HIFU combined with GnRH-a. The detection of serum CA125 and PGE2 may be used as an index to diagnose adenomyosis and evaluate the therapeutic effect of HIFU combined with GnRH-a.

## 1 Introduction

Adenomyosis, also known as intrinsic endometriosis, refers to the pathological changes caused by the invasion of endometrium (including gland and stroma) into the growth of uterine muscle layer [1]. Injection of gonadotropin-releasing hormone agonist (GnRH-a) is the main treatment of adenomyosis currently. The short-term effect of this drug is obvious, while the long-term use can cause low estrogen symptoms, and adenomyosis is easy to recover after withdrawal [2].

High-intensity focused ultrasound (HIFU) is noninvasive. It focuses on hyperacoustic energy on the target tissue or organ through injection *in vitro* and makes use of the thermal effect and cavitation effect produced by ultrasonic energy deposition to inactivate the lesion tissue, leading to the coagulative necrosis of lesions [3]. However, HIFU also has limitations, including skin toxicity, nerve, periosteal injury and intestinal injury, and other adverse reactions, and the symptoms may recur after treatment. It has been shown that HIFU combined with GnRH-a is more effective in the treatment of adenomyosis than HIFU or GnRH-a alone [4,5].

Carbohydrate antigen 125 (CA125) is a molecular glycoprotein, and it often appears in the epithelial tissue of cavities. As a kind of surface antigen in the mesothelial tissue and accessory middle renal tube epithelial tissue, it is highly expressed in epithelial tissues of cervix, pelvic peritoneum, fallopian, and endometrium [6]. Serum CA125 has been reported to be closely related to adenomyosis [7].

Prostaglandin E2 (PGE2) is a kind of vasoactive substance transformed by prostaglandins, which is considered to be an important inflammatory mediator and painkiller in the body [8]. High content of PGE2 in the abdominal cavity of sufferers with endometriosis may involve in the pathogenesis of endometriosis [9]. The changes of serum CA125 and PGE2 in sufferers with adenomyosis before and after treatment with HIFU combined with GnRH-a remain obscure.

The objective of this study was to investigate the clinical significance of the changes of serum CA125 and PGE2 in patients with adenomyosis before and after treatment with HIFU combined with GnRH-a.

## 2 Materials and methods

### 2.1 Clinical materials

This study was conducted in Suzhou Hospital of Integrated Traditional Chinese and Western Medicine, China, from August 2018 to December 2021. One hundred and sixty-five patients with adenomyosis who received HIFU combined with GnRH-a were selected as case group. Inclusion criteria were that sufferers with dysmenorrhea as the main symptom, and the degree of dysmenorrhea assessed by visual analog score was 4–10 points with or without menstruation; sufferers with adenomyosis diagnosed with pelvic magnetic resonance plain scan and contrast-enhanced examination, with the diameter of lesions greater than 3 cm; and sufferers who were willing to be treated with HIFU combined with GnRH-a; color Doppler ultrasound showed adenomyosis, and the focal point of transducer of HIFU equipment could reach the center of the lesion, no obstacle to communication.

Exclusion criteria were that lactating and pregnancy women; sufferers complicated with hypertension, diabetes mellitus, hepatic, renal, or cardiac dysfunction; sufferers who were unable to prone or fill bladder for more than 1 h; allergic to steroid drugs; sufferers with acute pelvic inflammation or chronic pelvic inflammation; sufferers with the overuse of steroid drugs recently; sufferers with enhanced magnetic resonance examination and anesthesia sedation taboo; sufferers with severe coagulation dysfunction; and sufferers who received other related treatments in the first 3 months before inclusion.

Sixty-five healthy women who underwent physical examination in this hospital at the same time were taken as normal control group. Case group was treated with HIFU combined with GnRH-a. Patients were administrated with HIFU ablation therapy with HIFU tumor therapy system. On the 1st to 2nd day of the first menstruation after HIFU ablation, the patients were subcutaneously injected with GnRH-a (leuprorelin acetate) 3.75 mg and then injected every 28 days for three consecutive courses. After the aforementioned treatment, the case group was followed up for 6 months from the first recovery of menstruation after treatment. At the end of follow-up, sufferers in the case group were divided into the effective subgroup and ineffective subgroup according to the clinical efficacy.

The criteria for judging the clinical efficacy of the case group were as follows: (i) remarkably effective: the symptoms such as dysmenorrhea and abnormal menstruation disappeared or basically disappeared, the shape of uterus was normal, and the volume of lesion was reduced by more than 70%; (ii) effective: the clinical symptoms such as dysmenorrhea and abnormal menstruation were obviously improved, the shape of uterus was basically normal, and the volume of lesion was reduced by 50–70%; and (iii) ineffective: the clinical symptoms and uterine size of the patients were not significantly improved, and the volume of the focus decreased by less than 50% or even increased [10]. The remarkably effective sufferers and effective patients in the case group were considered as effective subgroup, and invalid sufferers were considered as ineffective subgroup.

### 2.2 Detection method

The fasting venous blood of sufferers in the case group was collected before and on the third day of the last menstruation in the 6 months of follow-up. The fasting venous blood was collected in the morning on the day of physical examination in the normal control group. The level of serum CA125 was measured by automatic electrochemiluminescence immunoassay. The level of serum PGE2 was measured by enzyme-linked immunosorbent assay.

### 2.3 Statistical analysis

Data were processed using SPSS 25.0. Measurement data conforming to the normal distribution were expressed as mean ± standard deviation, compared between groups by independent samples *t*-test, and paired samples *t*-test was used for comparison before and after treatment within groups. Count data were expressed as *n* (%). *P* < 0.05 was statistically significant.


**Informed consent:** Informed consent has been obtained from all individuals included in this study.
**Ethical approval:** The research related to human use has been complied with all the relevant national regulations, institutional policies, and in accordance with the tenets of the Helsinki Declaration and has been approved by the authors’ institutional review board or equivalent committee.

## 3 Results

### 3.1 Comparison of demographic characteristic and serum indexes between case group and normal control group before treatment

There was no difference in age between the case group and the normal control group (*P* = 0.152). Serum CA125 and PGE2 levels in the case group were higher than those in the normal control group before treatment (both *P* < 0.001) (Table 1).

**Table 1:** Comparison of demographic characteristic and serum indexes between the case group and the normal control group before treatment

Index	Case group before treatment (*n* = 165)	Normal control group (*n* = 65)	*P* value
Age (years)	34.42 ± 5.19	35.46 ± 4.20	0.152
Serum CA125 (U/mL)	42.81 ± 3.64	17.95 ± 1.89	<0.001
Serum PGE2 (pg/mL)	263.16 ± 21.96	98.28 ± 10.33	<0.001

### 3.2 Comparison of serum indexes in case group pretreatment and 6 months after treatment

Serum CA125 and PGE2 levels in the case group 6 months after treatment were lower than those before treatment (both *P* < 0.001), as shown in Table 2.

**Table 2:** Comparison of serum indexes in case group pretreatment and 6 months after treatment

Index	Case group before treatment (*n* = 165)	Case group 6 months after treatment (*n* = 165)	*P* value
Serum CA125 (U/mL)	42.81 ± 3.64	36.82 ± 3.32	<0.001
Serum PGE2 (pg/mL)	263.16 ± 21.96	185.26 ± 17.52	<0.001

### 3.3 Comparison of serum indexes between effective subgroup and ineffective subgroup

After treatment, all sufferers in the case group were followed up for 6 months. Among 165 sufferers in the case group, 63 (38.18%) were remarkably effective, 85 (51.52%) were effective, and 17 (10.30%) were ineffective. The remarkably effective patients and effective patients in the case group were included in effective subgroup, namely, there were 148 cases in the effective subgroup. The ineffective patients were included in the ineffective subgroup, namely, there were 17 cases in the ineffective subgroup.

There was no difference in serum CA125 and PGE2 levels between the effective subgroup and the ineffective subgroup before treatment (*P* = 0.351, 0.284, respectively). Serum CA125 and PGE2 levels in the effective subgroup were lower than those in the ineffective subgroup 6 months after treatment (both *P* < 0.001) (Table 3).

**Table 3:** Comparison of serum indexes between effective subgroup and ineffective subgroup

Index	Point of time	Effective subgroup (*n* = 148)	Ineffective subgroup (*n* = 17)	*P* value
Serum CA125 (U/mL)	Before treatment	42.90 ± 3.67	42.03 ± 3.30	0.351
	At 6 months after treatment	36.37 ± 3.12	40.68 ± 2.46	<0.001
Serum PGE2 (pg/mL)	Before treatment	262.54 ± 22.50	268.58 ± 16.07	0.284
	At 6 months after treatment	182.37 ± 15.61	210.40 ± 12.59	<0.001

### 3.4 Magnetic resonance imaging (MRI) analysis of a typical case in case group

**Figure 1:**
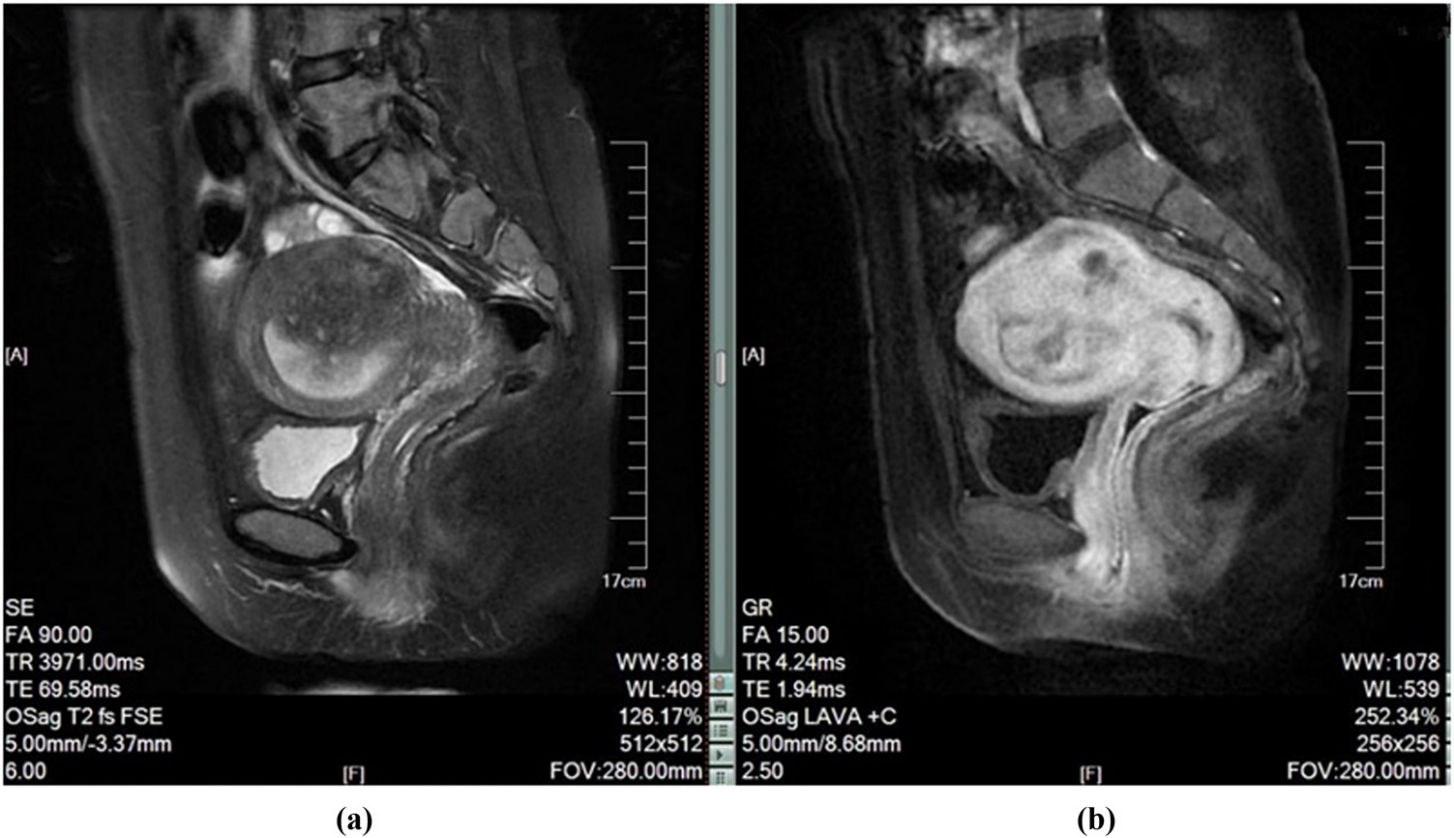
MRI image before treatment: (a) T2 sagittal MRI image and (b) enhanced sagittal MRI image.

The typical case in the case group was a 36-year-old female with adenomyosis. Before treatment, MRI plain scan and enhanced images were obtained (Figure 1(a) and (b)). Imaging features showed anteflexed and anteverted uterus, with a large nearly round mass in the posterior wall of the uterus measuring approximately 67 × 62 × 50 mm. The boundary was unclear, and the signal was uneven. Patchy high signals were seen in the T1W image, T2fs suppression showed mixed high and low signals, and DWI showed an iso-signal shadow. Enhancement showed progressive strengthening with uneven distribution and protrusion into the uterine cavity, indistinctly displayed in the binding zone. An elliptical abnormal signal was observed in the muscular layer of the uterine fundus, showing T1W equal signals, slightly low signals in T2fs suppression, uniform signals, clear edges, and uniform enhancement.

**Figure 2:**
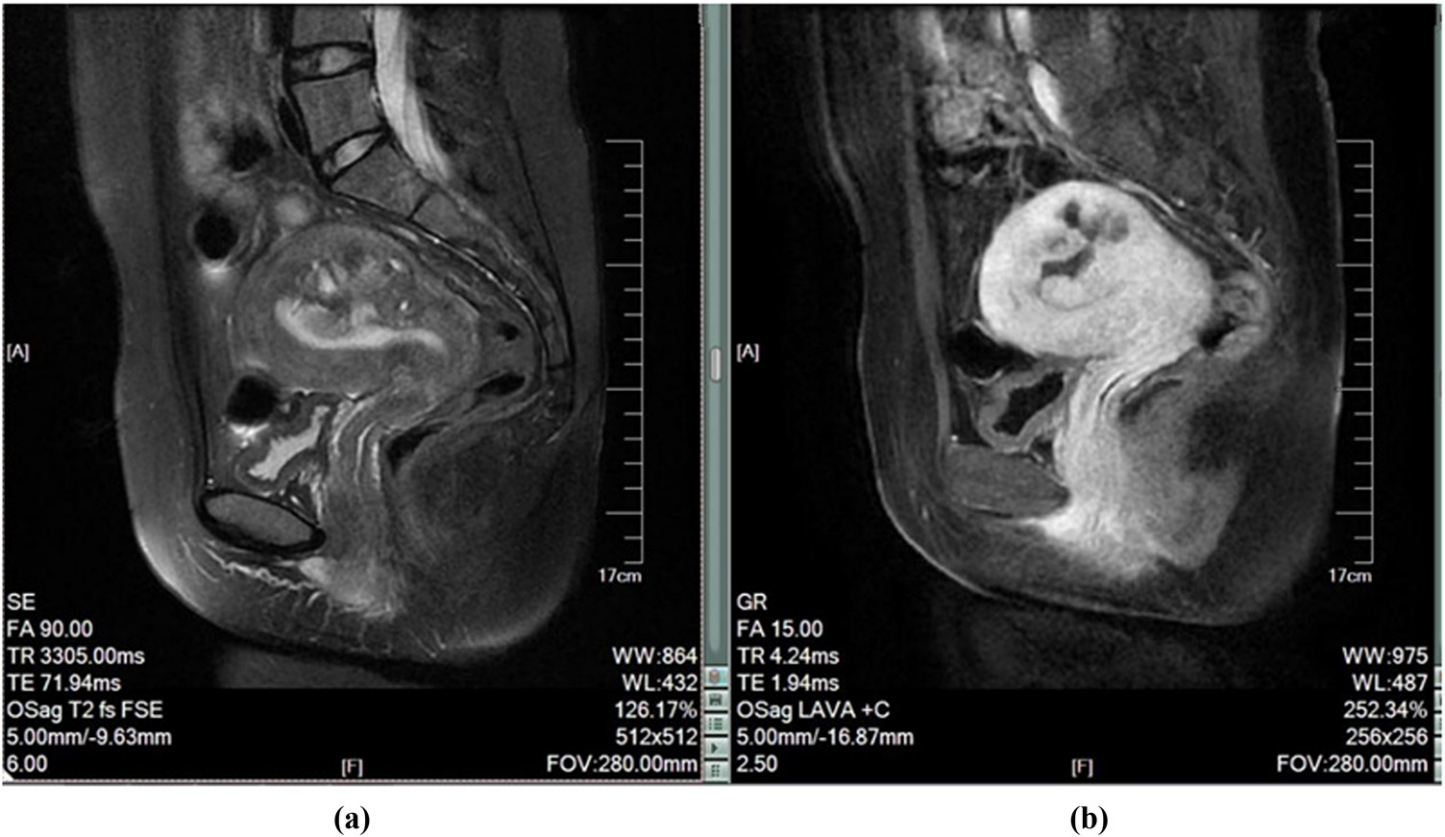
MRI image at 6 months after treatment: (a) T2 sagittal MRI image and (b) enhanced sagittal MRI image.

At 6 months after treatment, MRI plain scan and enhanced images were obtained (Figure 2(a) and (b)), showing an anteflexed and anteverted uterus with a large nearly round mass in the posterior wall of the uterus measuring approximately 48 × 48 × 48 mm. The boundary was unclear and the signal was uneven. Patchy high signals were seen in the T1W image, T2fs suppression showed mixed high and low signals, and DWI showed an iso-signal shadow. Enhancement showed a peripheral ring strengthening, protrusion into the uterine cavity, and indistinctly displayed in the binding zone. An elliptical abnormal signal was observed in the muscular layer of the uterine fundus, showing T1W equal signals, slightly low signals in T2fs suppression, uniform signals, clear edges, and uniform enhancement.

## 4 Discussion

Adenomyosis is a special type of endomyosis. The traditional diagnosis of adenomyosis mainly depends on clinical symptoms, physical signs, and B-ultrasound examination, which cannot help diagnose accurately and timely, so pathological examination is finally needed to confirm the diagnosis. Pathological examination is not suitable for early diagnosis due to invasive nature. Although nuclear magnetic resonance imaging is sensitive in the diagnosis of adenomyosis, the high cost limits its clinical applications [11]. Therefore, it is urgent to find a reliable marker or marker combination that can help in diagnosis and differential diagnosis for adenomyosis patients.

Expression of serum CA125 in sufferers with adenomyosis is relatively high [12]. However, it has been found that the diagnostic accuracy of serum CA125 alone in adenomyosis is limited [13]. PGE2 is an important inflammatory mediator and painkiller, which is related to inflammation, anaphylaxis, immunoreaction, and other pathological processes [14]. However, the value of serum PGE2 in the diagnosis of adenomyosis is still unclear.

We found that the levels of serum CA125 and PGE2 in the case group were higher than those in the control group before treatment, suggesting that the levels of serum CA125 and PGE2 in sufferers with adenomyosis are higher than those in normal women.

Many adenomyosis patients refuse to remove the uterus, so conservative treatment is particularly important. HIFU is a new conservative treatment [15,16]. GnRH-a is widely used in the treatment of gynecological diseases [17]. HIFU can inactivate the tissue of adenomyosis and cause coagulative necrosis, while GnRH-a can inhibit the pituitarium and keeps the estrogen in a low level, thus enhancing the curative effect [18]. We found that the levels of serum CA125 and PGE2 in the case group before treatment were lower than those after 6 months of follow-up. There was no difference in serum CA125 and PGE2 levels between the remarkably effective subgroup and the ineffective subgroup before treatment. The serum CA125 and PGE2 levels in the effective subgroup were lower than those in the ineffective subgroup after 6 months of follow-up. We found that the levels of serum CA125 and PGE2 in sufferers with adenomyosis were high before treatment, while their levels decreased significantly after treatment with HIFU combined with GnRH-a. The reason may be that HIFU combined with GnRH-a is effective in the treatment of adenomyosis, which can improve the condition of adenomyosis and reduce the expression of CA125 and PGE2.

It is worth mentioning that the follow-up duration of patients with adenomyosis treated with HIFU and GnRH-a in this study was short, and cases in the ineffective subgroup were limited. These factors may cause bias of the results in our research. Therefore, further well-designed studies with long follow-up period and large sample are needed to verify results in this research.

## 5 Conclusion

Serum CA125 and PGE2 may be involved in the development of adenomyosis, and their expression levels may be related to the prognosis of sufferers. Levels of serum CA125 and PGE2 in sufferers with adenomyosis decrease after treatment with HIFU combined with GnRH-a. Detection of serum CA125 and PGE2 may be used as an index to diagnose adenomyosis and evaluate the therapeutic effect of HIFU combined with GnRH-a.
